# Genomic sequencing in newborn screening: balancing consent with the right of the asymptomatic at-risk child to be found

**DOI:** 10.1038/s41431-024-01677-w

**Published:** 2024-08-12

**Authors:** Bartha Maria Knoppers, Ana Eliza Bonilha, Anne-Marie Laberge, Arzoo Ahmed, Ainsley J. Newson

**Affiliations:** 1 Centre of Genomics and Policy, Department of Human Genetics, Faculty of Medicine and Health Sciences, McGill University, Montreal, QC Canada; 2 McGill University Health Centre, Department of Human Genetics, Montreal, QC Canada; 3 Medical Genetics, Department of Pediatrics, CHU Sainte-Justine and Université de Montréal, Montreal, QC Canada; 4 Our Future Health, London, United Kingdom; 5 Sydney Health Ethics, Sydney School of Public Health, Faculty of Medicine and Health, The University of Sydney, Sydney, NSW Australia

**Keywords:** Ethics, Genetics

## Abstract

In this paper, we explore key aspects of the complex ethical and legal landscape surrounding consent in the context of incorporating genomic sequencing into existing newborn bloodspot screening programs. In particular, we consider the potential impact of genomic sequencing on the health rights of the child in relation to existing consent practices in newborn screening. We begin with an introduction to newborn screening programs and their population health goals. We then discuss public health ethics as a rationale underpinning newborn screening before turning to consent. We go on to describe seven current research projects on genomic sequencing in newborn screening and then introduce the ‘right of the asymptomatic at-risk child to be found’ as a useful concept to draw on when considering consent to newborn screening. We draw on this novel right to argue for the adoption of “appropriate consent” when it comes to certain uses of genomics in newborn screening. We contend that, for ‘virtual panels’ at least, appropriate consent proportionately balances the ongoing universality of newborn screening for important health conditions with an acknowledgement of the complex outcomes that bringing a complicated diagnostic technology into the screening domain will generate.

## Introduction

Newborn screening programs aim to enable earlier detection of serious conditions that can be treated or prevented in childhood. Newborn screening programs began in many countries in the 1960s and 1970s and initially targeted a small number of conditions, including phenylketonuria and congenital hypothyroidism [1]. Both conditions were widely recognized as serious, and screening tests and treatments were then newly available. Early diagnosis and treatment changed the natural history of the conditions from severe intellectual disability to neurocognitive development within the normal range.

As a population screening program, newborn screening has traditionally adhered to the 1968 Wilson and Jungner screening criteria, including that it should respond to a recognized need, that timely treatment is available and that there is agreement on who should be treated [2]. Furthermore, conditions screened should be considered serious health problems, with well-documented natural histories and a latent or early symptomatic stage that could be identified with the screening test.

However, in recent decades the expansion of newborn screening has arguably been driven by new technologies such as tandem mass spectrometry (MS/MS), and new treatments, as well as factors such as patient advocacy and industry influence [3]. The number of conditions included in newborn screening programs varies by country and context, but now typically includes up to 50–60 conditions, depending on where it is being provided [4–8]. New screening criteria have also emerged, including equity and access considerations in response to new and emerging genomic technologies [9], even before next-generation sequencing opened the door to using genome sequencing in screening.

Because genomic sequencing technologies enable screening for hundreds of genetic conditions in a single test, they may drive further expansion of newborn screening to include additional rare conditions, underpinned by new rationales such as avoiding diagnostic odysseys [10]. Research studies are underway in different countries to generate evidence for the validity, utility, and efficiency of genomics-led screening technologies in newborn screening programs (Table 1).

**Table 1:** Research projects investigating genomic sequencing in healthy newborns.

Project	Location	Sample	Number of genes/conditions	Recruitment/enrollment	Research consent process
BabyScreen+	Australia (Victoria)	Residual sample from dried blood spot	604 genes; severe but treatable childhood-onset conditions	Health care provider (obstetricians usually) approaches women during pregnancy (at 32 weeks). They are directed to an online educational platform (Genetics Adviser).	Written consent is obtained through the Genetics Adviser platform. Genetic Counselor hotline available for questions.
BabySeq 2	USA (Multisite)	Separate sample. May also collect a saliva sample.	604 genes associated with severe, early-onset, treatable conditions.	Recruitment through pediatricians, in the first year of life. Community engagement for enrolling underserved communities. Recruiting in community health centers. Participants are randomly assigned into two groups (genome sequencing or not).	Parents meet three times with the research team, and complete surveys at each time. Written consent is obtained
BeginNGS	USA (Rady Children’s Institute)	Residual sample from dried blood spot	411 rare, actionable disorders	During pregnancy in an obstetrics office or immediately after delivery in a hospital room.	Written informed consent from one or both parents.
Early Check	USA (North Carolina)	A different sample, taken at the same time as the blood spot	3 groups of conditions: (1) 180 treatable conditions (2) panel of about 30 diseases that have potential treatments, (3) genetic risk score for type 1 diabetes	Providers are not asked to recruit patients for Early Check. Recruitment through the state department. Can enroll babies up to a month after birth.	Existing online platform for education and written consent They can choose between the three different groups.
First Steps	Greece (Multisite)	A different sample, taken at the same time as the blood spot	500 genetic conditions where early-life intervention is available	Recruitment during pregnancy by OBGYN and in the nursery.	N/A
GUARDIAN Study	USA (New York)	Residual sample from dried blood spot	150 early-onset, treatable or intervention available, while 100 optional neurological with action available to treat some of the symptoms	Expecting parents can sign up for the study prenatally if they are more than 12 weeks pregnant.	Parents complete the study consent after the baby is born (up to 30 days after birth). Written consent is required
Generation Study	UK	Cord blood (primary sample) or heel prick (secondary sample when primary sample fails)	223 individual conditions caused by genetic changes in over 500 different genes. Principles for selected conditions available on website. Summary: Evidence of pathogenicity, high penetrance with important impacts on quality of life, early intervention can improve outcome, intervention is accessible to all.	Recruitment takes place in a hospital setting via a research team and trained clinical healthcare professionals. Expecting parents are made aware of the study after 20 weeks of pregnancy.	Prior to 36 weeks’ gestation, parents contacted by the site study team to have a conversation. Study consent form reviewed with a member of the site study team and consent decision recorded. Consent reaffirmed before sample collection on Day 0 as the baby is born.

While it may be argued that the introduction of genomic sequencing to newborn screening merely continues an existing trajectory of expansion, the implications of such expansion for consent to newborn screening remain under-explored. For example, both MS/MS and genomic sequencing bring with them the ability to ‘see but not report’ information. Both technologies also see the importation of a technology mainly used for diagnostic purposes in clinical care into a population health paradigm, with different goals and target groups. Yet unlike MS/MS, genomic sequencing information is likely to be stored for ongoing interrogation, including conditions beyond the targets of NBS [10, 11]. These factors are all relevant to how consent for the use of genomics in newborn screening should be designed and implemented.

With evidence regarding the use of genomic sequencing in newborn screening being generated, existing screening criteria remain relevant and applicable. The technological ability to detect conditions is not proof of a need for screening. Indeed, scientific evidence of improved diagnostic yield is not evidence of screening effectiveness. For example, effectiveness of the use of genomic testing in other domains (e.g. clinical genomic testing of unwell newborns in intensive care units) cannot be extrapolated to effectiveness of screening in healthy newborns as part of a screening program.

Any future implementation of genomics in newborn screening programs should ensure that parents or guardians can make autonomous decisions about such screening. A central tension in achieving this is that consent will become increasingly difficult due to the nature and complexity of genomic sequencing processes and potential results. As the number of conditions screened in newborns has increased since the introduction of MS/MS, consent has already evolved from a condition-based information paradigm to one based on information about groups or types of conditions being screened. The integration of genomics will further push the consent process towards general information and rationale about the types of conditions being screened.

In our view, genomic newborn screening has yet to meet the key challenge of articulating how consent should occur. We argue that, at least for some forms of genomic newborn screening such as virtual panels, a modified form of ‘appropriate consent’ [12] is a suitable approach. We anchor this approach in a balancing of a novel right of the asymptomatic at-risk child to be found (through screening) with how consent to genomic newborn screening should be implemented. As we argue, the initial ‘case finding’ on which population screening is based should not be undermined by complexity of consent. This also provides a reason for caution in the unconstrained use of genomics in first-line newborn screening, as such wider uses will necessarily add complexity to consent and may risk the right of the child to be found.

### Newborn screening and public health ethics

A premise for our position is that the use of genomics in newborn screening is best classified as population (or public) health rather than clinical care. It comprises a single screen offer and is aimed at a specific asymptomatic population, rather than being offered to certain individuals in light of a particular clinical presentation or family history. Variant calling with genomic sequencing draws on population level knowledge rather than using a participant’s family history. If genomic sequencing is used in a uniform way (with the same gene/variant list used for all screening participants), then together with other components such as pathways for screen-positive newborns, genomic sequencing would meet criteria to be termed a population screening program [13, 14].

As a population screening program, newborn screening (and thus the use of genomics in newborn screening) needs to be considered against a background of public health ethics [15]. Scholars have begun to recognize the value of public health ethics in newborn screening [16] and in genomic population screening [17] more generally. The conceptual tools and ethical approaches that public health ethics brings allow us to look at wider social and structural factors, and to consider issues that arise at a collective or community level as well as for individuals. Concepts and values such as solidarity, reciprocity, caring, equity, identifying common goods and promoting interpersonal connectedness can be given greater attention under a public health ethics framing [15, 18]. Further, framing the use of genomics in newborn screening as a public health ethics problem will allow us to consider the benefits and burdens of alternative approaches to consent with other aspects such as public health goals, fairness [18] and (we argue below) the population health right of the asymptomatic at-risk child to be found.

A public health ethics approach is also well aligned to newborn screening as a population health initiative, one that aims to improve the health of a population (as well as the health of individuals within that population). It aims to *prevent* ill health rather than treat it and will not succeed unless there is widespread ‘buy in’ and case-finding. Within this approach, we can start to see the place and nature of consent in genomic newborn screening, and the tensions some approaches to consent will give rise to when implemented at population scale. While consent will serve an important role in citizen agreement to genomic newborn screening programs, it also needs to be sustainable to deliver at population scale, with attention to equity, health literacy, and appropriate resource use. If a consent process will undermine the right of asymptomatic at-risk children to be found through newborn screening, then this provides good reason to limit genomic sequencing to a well curated panel undertaken with appropriate consent.

### Newborn screening and consent

Consent embodies the freedom of competent persons to choose. In the context of clinical care, it expresses the voluntary and ongoing choices made by individuals who are being offered treatment in their best interests. Here, the standard of consent is typically fully informed consent. For consent to be informed, communication of risks, benefits and alternatives are essential. Research, in contrast, seeks the acquisition of generalizable knowledge. In this context, communication obligations are generally heightened due to the greater risks and the lack of, or unknown and undefined, personal benefit. In population health programs such as vaccination or screening, general information is provided to a target population on the overall goals and benefits of the screening initiative, as well as its modalities.

Generally, consent (whether to clinical care, research or population health) can be verbal or written but it should be context specific. Implied consent means that the relevant information has been communicated and received and that the recipient has not objected, opted-out, or, withdrawn.

Whilst clinical care generally requires fully informed consent, population health programmes such as newborn screening vary in the type of consent they obtain. Although newborn screening in countries like the United Kingdom or Australia requires a more explicit form of (verbal or written) parental consent [19, 20], the process varies. Participation is even mandatory in some settings, such as certain countries in Latin America [21]. In the United States, some states have legislated routine screening so that samples from babies can be collected and tested without explicit parental consent, although parents should be notified and given the chance to opt-out on religious or other grounds [22]. A 2014 study of newborn screening in Canada found that parental consent for newborn screening is typically implied rather than explicit [23]. For example, in Quebec, a nurse obtains verbal consent from the parent and documents it on the laboratory requisition form. Opting out of the program requires a parental signature [24]. In a 51-country European study of newborn screening, participation was mandatory only in Italy while 64% of countries required consent, meaning that at least verbal consent was sought [25] (Personal communication (Loeber) Dec. 20, 2023). Indeed, in another 27-country study European newborn screening regulatory landscapes, only 11 required written informed consent [26].

In short, most countries globally use an implied consent model for newborn screening. Information about screening is usually provided during prenatal visits or as part of standard care at birth. Newborn screening is viewed as a routine procedure, with parents not formally asked to consent (or having no recollection of having given it), or the test proceeds unless parents opt-out [27, 28]. Research undertaken in England, for example, showed that most parents felt newborn screening was not presented as optional [29].

That said, there is consensus that parents should be informed about newborn screening and its stated goals. In most countries, there are points in time such as birth or (preferably) during prenatal care where potential parents are informed about newborn screening. Once an asymptomatic at-risk (‘screen positive’) newborn has been identified, diagnostic confirmatory testing, repeat sampling, storage, and use for research, all require separate and specific parental consent.

When it comes to the use of genomics in established newborn screening programs, tensions in consent emerge. One consideration is that the form of testing may be similar to that used in clinical genomic testing. Many of the test processes and pipelines, and high-level variant calling, will be the same, as will the type of information the test may generate. Some proposed models for genome sequencing in newborn screening have considered it as a form of precision medicine – and as such would incorporate extensive and/or dynamic consent protocols [30]. If the eventual model for genomics in newborn screening involves whole genome sequencing and a wide scope of information provision, then traditional approaches to consent for screening may not be appropriate. Instead, consent processes would need to be adapted to reflect the type and amount of information that genomic screening may reveal. In other words, an approach to consent more akin to that seen in clinical care would need to be considered.

However, a second consideration – that pulls in tension with the first – is that the use of genomics in newborn screening is *not* clinical care and would not be feasible to provide as such. An offer of a public health intervention brings with it an imputed message that the intervention is worthwhile taking up, and ‘case finding’ is a key goal of screening. As a result, consent in the context of a population health program tends to not be as granular or information-rich as it would be in clinical care or research. Of course, confusion can easily arise here, because in the use of genomics in newborn screening, we will see the transplantation of a clinical diagnostic intervention to a population health setting. Furthermore, most of the uses of genomics in newborn screening are currently occurring under research protocols, and so consent to participation has necessarily followed regulatory requirements for research, leading to detailed consent protocols. As a result, careful attention needs to be given to how consent can be considered and operationalized as and when genomics is introduced as a first-line test in newborn screening.

### Newborn screening and genomic sequencing

Over a decade ago, the possible expansion of newborn screening to include genomic sequencing was labeled as potentially “discordant, disorganized and disruptive” [31]. At that time, the cost of sequencing, ethical, legal and social issues, and the possible impact on universal accessibility, were considered to justify the continued importance of targeted approaches [32, 33]. Public views on genomic newborn screening revealed “the possibility of reduced participation” and raised concerns over challenges to “the moral authority” of newborn screening to “ensure population benefits” [23]. Others considered that the “newborn period was not the right time for such testing” [34, 35].

Today genomics remains subject to several important limitations, which suggest ongoing caution in its wide application in population health [15]. It also remains true that “the complexity of genome sequencing, the variety and uncertainty of potential results, the broad implications of those results, and the elevated expectations of personal benefit….create(ed) new or amplified challenges for informed consent” [36].

Nevertheless, several pilot research projects to consider the validity and utility of genomic sequencing for screening purposes in healthy newborns are underway or have recently concluded. Two completed projects (*NC Nexus and BabySeq 1*) used Whole Exome Sequence (WES) to look at up to 822 and 954 genes respectively. In Table 1, we detail seven further ongoing or imminent research projects. All propose the use of genomic sequencing, but undertake analysis using virtual panels of conditions. The information in Table 1 was collected during the ICoNS 2023 Conference attended by one of the authors (AEB) and supplemented with information obtained from project websites (Supplementary Table 1).

As they are research, these projects necessarily obtain explicit parental consent for genomic screening, separate from any consent process for existing newborn screening programs. The number of conditions screened in these research projects ranges from 180 to 604. Consensus on the selection of conditions for screening has proven challenging, since different principles are relied upon for the inclusion of conditions in different research projects [37]. However, there are moves towards global consensus [38]. As discussed, the availability of treatment during childhood has traditionally been a central criterion for inclusion of a condition in newborn screening. Two studies (*Early Check and Guardian*) also offer an optional panel of early onset conditions with no treatment available, but with possible preventive interventions during childhood. This represents a departure from traditional newborn screening criteria and signals what may also happen with future genomic sequencing in newborn screening programs. One research project (*Early Check)* also offers an optional polygenic risk score for Type 1 diabetes.

Explicit, written parental consent for these research projects is typically being obtained and recorded during pregnancy. While the preference was to obtain consent during the prenatal period, most programs also facilitate enrollment after birth. In five of the studies, health care professionals, such as obstetricians, nurses, and pediatricians, approached parents for project enrollment. In two programs (*BabyScreen+* and *EarlyCheck)*, interested parents are directed to an online educational platform where they could provide consent. *BabyScreen+* also offers a “hotline” for parents who wish to speak with a genetic counselor if they have any questions. Decision aids and/or educational materials are offered by all programs.

For the return of results, low risk (negative) results are accessible through an online platform or conveyed via a letter to parents. In the case of positive (high risk) results, genetic counselors are typically involved. The number of positive results is in proportion to the number of genes/conditions tested. When testing for hundreds of conditions, the number of children who will need to be evaluated for diagnostic confirmation and early management will increase dramatically, which will also require additional resources for counseling and to obtain consent to diagnostic follow-up.

The consent approaches in these research projects (Table 1) revolved around key aspects such as the timing of parental enrollment or the necessity of pre-consent counseling (e.g., utilizing an online platform vs. recruitment through a health care provider or research team member). Further challenges to consent include: the complexity of genomic information; the issue of whether variants of uncertain significance (VUS) are reported; the variable penetrance and expressivity of genotypes; the practical challenges of requiring written consent; future possible duties to re-interpret results and re-contact families; feasibility; and the limited variable familiarity of healthcare professionals with genomics knowledge to enroll and follow parents in such programs.

### Newborn screening and the right of the asymptomatic at-risk child to be found

What is missing from existing research consent discussions is any reference to the most relevant party: the newborn who is screened. Deference to parental authority, permissions, and duties are rightly recognized in both the research and clinical settings. But newborn screening is primarily about finding asymptomatic at-risk newborns and only offering follow-up to those actually at-risk. This search could be considered a legally actionable duty since the adoption of the UN international Convention on the Rights of the Child (1989), signed and ratified by 196 States Parties [39]. It states (art. 3) that the “best interests of the child shall be a primary consideration” in all actions concerning children. Amongst other rights, it also upholds: the right to be heard (art. 12); the right to privacy (art. 16); and the right to the highest attainable standard of health (including pre- and postnatal care (art. 24)). Article 24 does not create a right to be born healthy but rather a legal duty to maximize the potential for children to enjoy this right. Developing preventive health care is also included [40]. Hence, coupled with the challenges of communicating what genomic sequencing is and its possible benefit in newborn screening is the legal obligation to respect children’s actual and future health rights and needs.

Newborn screening is population-based and is usually the responsibility of publicly funded entities to oversee and implement. Additionally, under the parens patriae doctrine, States are obliged to protect the vulnerable. Failure to provide medical care or take preventive health measures can be considered child medical neglect under youth protection legislation [41]. To this end, we contend that these rights can be used as a basis to put forward a novel ‘right of the asymptomatic at-risk child to be found’ in order to underpin the rationale within all newborn screening programs to identify children with actionable conditions. This right to be found may be especially pertinent for previously excluded (rare) conditions, not least because such conditions may only be identifiable using genomic sequencing methods.

Of course, the evidence threshold to include conditions in genomic newborn screening should be maintained, i.e., the available evidence must demonstrate effectiveness of the proposed intervention/treatment. This right also extends only to situations where the newborn will be truly ‘found’ and would not include situations such as returning variants of uncertain significance, or carrier status. Thus, the right to be found can be used to inform how we think about consent to genomic newborn screening as a program.

### Genomic newborn screening and “appropriate” consent

In any move to genomic newborn screening, the status of genomic newborn screening as a public health initiative and the relevance of the right of the asymptomatic at-risk child to be found make managing consent a complex exercise. Several questions remain unaddressed: Is fully informed consent to genomic newborn screening feasible or desirable in a screening context? Should the potential range of results be the focus, or something else such as the program rationale and possible outcomes? Are today’s healthcare professionals or departments of public health sufficiently “genomically” literate?

A central consideration is how genomic sequencing is used in the context of newborn screening. If it is implemented as a new tool to detect conditions that meet existing newborn screening criteria, then the number of additional conditions and types of results will be limited since they will require evidence for treatment effectiveness. If however the integration of genomic sequencing leads to broadening of the scope of newborn screening to include conditions that are not treatable, or if it includes disclosure of late-onset conditions or carrier status, then the consent process will need to be reviewed substantially.

Two decades ago, Elias and Annas proposed the concept of ‘generic consent’ to genetic screening [42]. Here, consent would encompass broad concepts and “common denominator issues”, with the aim to avoid providing too much or irrelevant information. More recently, the PROMICE novel framework for pediatric clinical genomic testing suggests refocusing ethical inquiry on whether consent is appropriately informed, not on whether it is “fully” informed [12]. This is especially important since “appropriate” consent emphasizes the promotion of subjective well-being. Under this model, “the described goal of promoting well-being could be given greater relevance than promoting autonomy, since it is proxy decision-making” [12]. Moreover, “promoting autonomy in most cases of pediatric (WGS [whole genome sequencing]) primarily aims to preserve the autonomy of the proxy decision-makers, rather than that of the children themselves” [43]. Although advanced for pediatric clinical sequencing, we argue that appropriate consent is also relevant to screening because we can consider it in light of the right to the asymptomatic at-risk child to be found.

Further support for an approach like PROMICE is provided by studies that have already begun to show that even for clinical genetic testing integrated into adult healthcare, “full disclosure of all information about genetic testing would simply be impossible…(A) balance must be sought between comprehensiveness and comprehensibility” [44]. Guidance from The Netherlands suggests providing a simple, brief explanation of the institutional policy [45]. A recent survey of the applicability of the US-based CADRe (Consent and Disclosure Recommendations) framework found general acceptance of a targeted, pre-test discussion approach, with the communication of core informed consent concepts, that is, of the “minimum” critical components [46]. In any case, pre-screen education and engagement requires appropriate training and clear expectations for health professionals involved in discussing newborn screening with parents and obtaining consent [28].

Consent for population-scale genomic newborn screening programs could be based on the provision of appropriate, publicly co-designed, pre-screen information to both promote general understanding as well as the chance to reflect on the implications of screening. Consent should be multi-modal and meet the needs of diverse parents through effective, public pre- and post- screening information tools. Post-screening counseling for screen-positive newborns and explicit parental consent for any diagnostic testing or to research would still, of course, be required. A PROMICE-like approach to consent to genomic newborn screening arguably balances the feasibility necessary for a population health paradigm with clear communication of the rationale behind the panel of conditions to be reported. If a clearly defined and well justified (on screening principles) panel is adopted, then appropriate consent to such a panel-based screening test could be in the best interests of children (both as individuals and as part of a target population), including their right to be found through screening. However, appropriate consent would be neither suitable nor sufficient for newborn screening programs if they are contemplating activities such as open-ended whole genome sequencing (with no virtual panel) because the inherent complexity of these activities could not be accommodated. Appropriate consent may also not be feasible if genomic newborn screening is to routinely store data for future research, or reanalysis, although these possible limits are beyond the scope of this paper to debate.

## Conclusion

Newborn screening seeks to identify at-risk newborns via a public health program overseen by public or state health authorities. By its very nature, programmatic screening affects the form of consent that will be feasible when enabling the informed and voluntary participation of parents. Both the nature of communication and informed and voluntary participation are based on public understanding of the particular public health nature of newborn screening. But, even if limited to panels of conditions, the use of genomic sequencing in newborn screening necessarily increases the volume and complexity of screening data.

Along with the criteria of seriousness and treatability of the conditions being screened for, it is important to remember that the “benefits to the child” criterion in newborn screening dates to the 1968 WHO Wilson and Jungner principles and their later expansion. The health rights of the asymptomatic but at-risk newborn can arguably only be met via population screening.

We have argued for a balancing of a novel right – the right of the asymptomatic at-risk child to be found – with the complexities of consent when considering the use of genomics in newborn screening. We claim that appropriate consent to the use of genomics in newborn screening will require new and ongoing public communication as to the precise goals of universal screening of all newborns. Communication of the healthcare implications of screening and the choices that will follow should be specific to this population programmatic context, as well as being in the actual and future interests of children.

Under the principle of proportionality, the benefits of newborn screening should outweigh the risks [47]. Rigorous evidence for the introduction of genomic sequencing into newborn screening is still missing [48], and the seven research projects we have highlighted illustrate the complexity involved in generating this. At present, consent to newborn screening is at a crossroads: there is a pull to expansion of newborn screening, but at the same time this may risk the universality of screening due to consent complexities. Our argument allows for the introduction of genomics to newborn screening while retaining screening principles and detecting the things that screening has always been intended for.

Newborn screening at a population level is not primarily about individuals, parents, or families (although they should be engaged with), but rather about finding the at-risk child. If all these elements are achieved and finding asymptomatic at-risk newborns comes first, we suggest that genomic newborn screening need not be on a “collision course with public health ethics” [18] as not only will the form of consent provided be appropriate to this context, most importantly the health rights and interests of all asymptomatic at-risk newborns will be promoted and protected.

## Supplementary information

Supplementary Table 1
