# Supplementary material for: Genomic sequencing in newborn screening: balancing consent with the right of the asymptomatic at-risk child to be found
Source: Eur J Hum Genet. 2024 Aug 12;33(2):182–8. doi: 10.1038/s41431-024-01677-w (PMC11840138; doi:10.1038/s41431-024-01677-w)
Supplement: Supplementary file 1 — Supplementary Table 1 [file 41431_2024_1677_MOESM1_ESM.docx]

Supplementary Table 1: Websites of research studies involving genomic sequencing in newborns

# Supplementary Information to: “Genomic Sequencing in Newborn Screening: Balancing consent with the right of the asymptomatic at-risk child to be found.”

*European Journal of Human Genetics*

Authors: Bartha Maria Knoppers, Ana Eliza Bonilha, Anne Marie Laberge, Arzoo Ahmed, Ainsley J. Newson

| **Study name** | **Institution/Host** | **Website** | **Date accessed** |
| --- | --- | --- | --- |
| BabyScreen+ | Murdoch Childrens’ Research Institute | <https://babyscreen.mcri.edu.au/> | 19 January 2024 |
| BabySeq 2 | clinicaltrials.gov | <https://clinicaltrials.gov/study/NCT05161169> | 31 December 2023 |
| BeginNGS | Rady Children’s Institute for Genomic Medicine | <https://radygenomics.org/begin-ngs-newborn-sequencing/> | 19 January 2024 |
| Early Check | RTI International | <https://portal.earlycheck.org/> | 19 January 2024 |
| First Steps | Fabric Genomics and PlumCare | <https://www.firststeps-ngs.gr> | 19 January 2024 |
| GUARDIAN Study | Columbia University & New York State Department of Health | <https://guardian-study.org/overview/> | 19 January 2024 |
| Generation Study | Genomics England | <https://www.genomicsengland.co.uk/initiatives/newborns> | 19 January 2024 |
